# Proposed Plan for Medical Reform

**Published:** 1877-12

**Authors:** M. W. Wood

**Affiliations:** Asst. Surg. U. S. A. Fort McKinney, Wyoming Territory


### Correspondence.

### PROPOSED PLAN FOR A MEDICAL REFORM.

*To the Editor of the* MEDICAL JOURNAL AND EXAMINER.

The plan which I would propose has for its object an association of physicians and surgeons, somewhat similar to the Royal Colleges of Physicians and Surgeons of London, Edinburgh, and Dublin—for the purpose of elevating the tone of the profession, stimulating earnest, careful study and original investigations—for the purpose of benefiting pecuniarily and medico-socially its members—and for the purpose of enabling the profession, and more especially the laity, to discriminate between the charlatan, the drone and the mass of workers of the profession.

Under this plan there would be necessary the organization of a new medical society, which should require the candidate for membership to satisfactorily pass such examination as shall be prescribed on the fundamental principle and outlines of the several branches of medical science; and on such extra-professional topics in the collateral sciences and general information as may be determined upon.

I would also propose that there be established, as a further honor for those entitled to such distinction, a Fellowship in this society—a Fellowship difficult to obtain, to be regarded as an honor conferred, as a prize to strive for, and one to which (as the profession is now constituted) only the best men could aspire.

To accomplish this I would have the matter carefully thought over by the best men in the profession—then have a number of these sign a call, to be sent to each member of the regular profession in each city, and to such outside physicians as would be thought likely to attend, for a meeting to discuss and adopt a preliminary plan. The meeting to be called to order by some one who is a leader, who would in a few well-chosen words shadow forth the outlines of the scheme, and thus prepare the way for proceeding to a permanent *preliminary* organization. Then elect a Court of Examiners, and, through a *strong* committee, adopt a plan and scope of examination for the purpose of procuring a number of charter members, (say about 20) for the future medical society—the preliminary organization then to be considered as having accomplished its work. The *twenty* should now proceed to organize, elect officers, adopt a Constitution and By-laws, get an act of incorporation, and then zealously guard the interests of the profession.

After a period of *not less* than six months, let the members beget, as the charter members were begotten, about fifteen charter-fellows, who should also be incorporated, and grant diplomas.

I would authorize the Fellows to append to their professional signatures as an additional title, the initials of the name of the society.

I would *in the strongest and most positive terms* prohibit the conferring of *membership* upon any one, except those regular practitioners of medicine who have, in the regular way, secured the approval of the Court of Examiners; and just as strenuously prohibit the conferring of Fellowship upon any one, except such members as have in the regular way secured the approval of the Court of Examiners for this advancement; and I would have this secured by legislative enactment in the charter.

With a view to the proper remuneration of the Court of Examiners for their services, and for the purpose of placing the organization upon a solid financial basis, (and this I believe to be one of the strongest cements for holding such bodies together and to the mark) I would make fifteen ($15.00) dollars the minimum fee for the membership, and twenty ($20.00) dollars the minimum fee for the Fellowship.

I would have all of the examination papers in the case of each candidate sealed and carefully preserved, only to be opened, if desirable, by a two-thirds vote of all the members of the society.

I would make the limits of the society commensurate with the boundaries of the State, with a view to its early recognition by the State legislature, as authorized to exercise a censorship over medical professional matters within the State.

I would set the standard high from the first, certainly high enough to secure a selection of *not more* than sixty per cent. of the first year's candidates, and that very few might find the requirements easily satisfied.

I may be over sanguine, but I believe that the times are ripe for a movement of this kind, and that in several of the States the workers are ready to wheel into line as soon as some State has taken the initiative; and I furthermore believe that to the pioneers in a much-needed work of this kind due honor and credit will be gratefully accorded. I also believe that ere *ten* years would have passed, this membership and Fellowship would be rocognized and properly appreciated by the people, and be demanded by them as a qualification from aspirants for professional positions or for the brightest smiles of public favor.

M. W. Wood, *Asst. Surg. U. S. A.*

Fort McKinney, Wyoming Territory,
Oct. 17th, 1877.

